# High shear stress enhances endothelial permeability in the presence of the risk haplotype at 9p21.3

**DOI:** 10.1063/5.0054639

**Published:** 2021-07-26

**Authors:** Evan L. Teng, Evan M. Masutani, Benjamin Yeoman, Jessica Fung, Rachel Lian, Brenda Ngo, Aditya Kumar, Jesse K. Placone, Valentina Lo Sardo, Adam J. Engler

**Affiliations:** 1 Department of Bioengineering, University of California, San Diego, La Jolla, California 92093, USA; 2 Department of Neuroscience, The Scripps Research Institute, La Jolla, California 92037, USA; 3 Biomedical Sciences Program, University of California, San Diego, La Jolla, California 92093, USA; 4 Sanford Consortium for Regenerative Medicine, La Jolla, California 92037, USA

## Abstract

Single nucleotide polymorphisms (SNPs) are exceedingly common in non-coding loci, and while they are significantly associated with a myriad of diseases, their specific impact on cellular dysfunction remains unclear. Here, we show that when exposed to external stressors, the presence of risk SNPs in the 9p21.3 coronary artery disease (CAD) risk locus increases endothelial monolayer and microvessel dysfunction. Endothelial cells (ECs) derived from induced pluripotent stem cells of patients carrying the risk haplotype (R/R WT) differentiated similarly to their non-risk and isogenic knockout (R/R KO) counterparts. Monolayers exhibited greater permeability and reactive oxygen species signaling when the risk haplotype was present. Addition of the inflammatory cytokine TNFα further enhanced EC monolayer permeability but independent of risk haplotype; TNFα also did not substantially alter haplotype transcriptomes. Conversely, when wall shear stress was applied to ECs in a microfluidic vessel, R/R WT vessels were more permeable at lower shear stresses than R/R KO vessels. Transcriptomes of sheared cells clustered more by risk haplotype than by patient or clone, resulting in significant differential regulation of EC adhesion and extracellular matrix genes vs static conditions. A subset of previously identified CAD risk genes invert expression patterns in the presence of high shear concomitant with altered cell adhesion genes, vessel permeability, and endothelial erosion in the presence of the risk haplotype, suggesting that shear stress could be a regulator of non-coding loci with a key impact on CAD.

## INTRODUCTION

Heart disease remains the leading cause of death in the United States^1^ and, in particular, coronary artery disease (CAD) is the most prevalent form worldwide.^2^ While significant risk for CAD is associated with lifestyle, there is growing recognition that genetics has a profound influence on risk. Genome wide association studies (GWASs) have identified a number of single nucleotide polymorphisms (SNPs) within the 9p21.3 locus with extremely high correlation to increased risk of acquiring CAD and myocardial infarction.^3,4^ This region contains an antisense noncoding RNA called *ANRIL (CDNK2B-AS1)* and borders the CDKN2A and CDKN2B genes,^5,6^ whose function is not well understood. Notably, this particular locus is found only within close evolutionary relatives of humans, e.g., chimpanzees and Rhesus macaques; it is not found in commonly studied rodent species,^7,8^ and hence orthologous knockout studies of the syntenic mouse region^9^ yielded results with unclear human relevance. Given the challenges involved in human and primate research, patient-derived induced pluripotent stem cells (iPSCs) offer a suitable alternative means of interrogating the 9p21.3 CAD risk locus effects at the cellular level.^10^ Indeed, recent evidence suggests that the haplotype's presence significantly impairs the function of iPSC-derived cardiovascular progeny.^11,12^

The process of CAD-associated injury and plaque buildup within arterial walls involves several different cell types,^13^ including resident endothelial cells (ECs) and smooth muscle cells as well as immune response cells such as macrophages. With the presence of stressors within the blood, excessive tunica intima permeability from leaky tight junctions leads to fatty build up, expanded foam cell presence, and smooth muscle invasion, which decrease lumen area and restrict blood flow.^14^ While CAD progression is well studied, how this downward cascade of vessel health begins in relation to endothelial cell dysfunction is less understood. ECs reside in the innermost layer of the blood vessel, separating the lumen from the vessel wall and regulating nutrient and molecular transport beyond the vessel. In addition, ECs also are critical in mediating macrophage attachment and entry into the blood vessel via extravasation, which can exacerbate vessel injury.^15^ Like their cardiomyocyte counterparts,^12^ external factors—either chemical or physical—can influence how ECs are able to fulfill these functions.^16^ For example, EC barrier function is susceptible to inflammatory cytokines, notably Tissue Necrosis Factor alpha (TNFα) and Interleukin 6 (IL-6), which are typically present in cases of injury and inflammation.^17^ Hemodynamic shear stress also influences EC function.^16,18^ For example, laminar shear stress as well as low magnitude pulsatile flow are atheroprotective.^18^ However, disturbed or turbulent flow increases inflammation and cellular damage,^19^ in which high, pathological shear stress can reverse beneficial effects due to increased blood parameters of pressure, flow, or vessel tightening.^20^

The myriad of genetic, biochemical, and physical effects on ECs demonstrate a complex environment whose holistic effects are not fully understood. To better understand the role that the 9p21 region has on EC physiology and regulation of vessel integrity, we characterized iPSC-derived EC^21^ morphology and function in the presence of the risk haplotype. To introduce fluidic stress effects into the equation, we built a 3D microfluidic device comprised of an iPSC-derived EC microvessel within a collagen scaffold, and determined laminar shear effects on the EC microvessel, its permeability, and its structural integrity with relation to the 9p21 risk haplotype presence. These observations further suggest that the interaction between the 9p21 risk locus and environmental stress represents a key factor in CAD onset and progression.

## RESULTS

### 9p21.3 CAD risk haplotype does not impair iPSC-EC differentiation

**FIG. 1.**
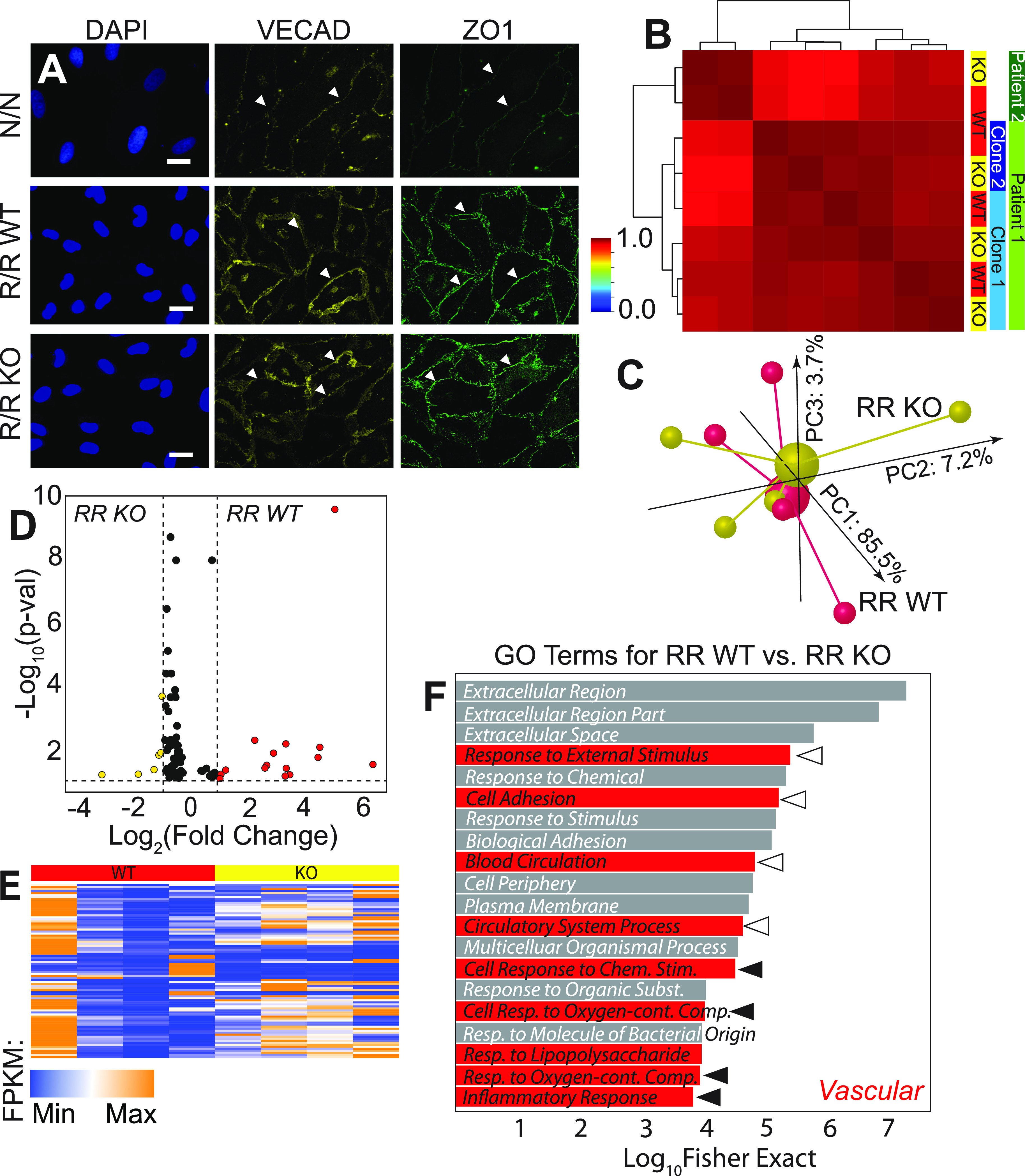
9p21 CAD risk haplotype does not impact EC commitment but does prescribe some transcriptome differences. (a) Images of nuclei (DAPI; blue), VE-cadherin (yellow), and ZO1 (green) for cells of the indicated haplotypes. Arrowheads indicate positive staining of proteins at the cell-cell junctions. Scale bar is 10 *μ*m. (b) Pearson's correlation plot is shown for patient iPSC-derived ECs based on the whole transcriptome sequenced from the patients, clones, and haplotypes indicated at right. Dendrogram indicates sample clustering; the color map is plotted between 0 and 1 as consistent with other figures. (C) Independent of patient and clonality, a 3D PCA plot is shown for transcriptomes from R/R WT (red) and R/R KO (yellow) ECs. Individual samples are shown as smaller spheres, whereas sample averages are indicated by a larger sphere noting its linkage via identically colored lines. The contribution of each PCA axis is noted alongside the axis itself. (d) Volcano plot and (e) heatmap of 86 DEGs for transcriptomic comparisons of R/R WT (red) and R/R KO (yellow) ECs with p < 0.1. Colors indicate in which haplotype were the genes upregulated. Color map also indicates the strength of expression in terms of fragments per kilobase of transcript per million (FPKM) mapped reads. (f) Top 20 gene ontological terms are plotted with their statistical significance and colored based on their association to cardiovascular stress; open and closed arrowheads correspond to ontologies related to blood flow and forces and to inflammation, respectively.

iPSCs carrying the homozygous risk haplotype (R/R assessed by genotyping at rs1333049, rs2383207, and r10757278), the knockout isogenic lines edited by TALENs (R/R KO), or those carrying the non-risk haplotype (N/N)^11^ were verified for their pluripotency via staining of Sox2, Nanog, and Oct4 [Fig. S1(A)], for which all lines and clones showed similar positive expression. The differentiation of iPSCs to ECs—through mesoderm and subsequently endothelial specification^21^ [Fig. S1(B)]—resulted in endothelial populations of 95+% purity via vascular endothelial cadherin (VE-Cadherin) expression across single non-risk, risk wildtype, and risk knockout lines [Fig. S1(C)] when assessed by flow cytometry. Moreover, staining for junctional proteins ZO1 and VE-Cadherin showed appropriate localization [Fig. 1(a)] and no noticeable morphological differences in circularity independent of the haplotype (Fig. S2). To ensure that basic EC lipid uptake was not affected by haplotype, iPSC-ECs were assessed for acetylated-LDL uptake, and we found that population percentage uptake was not impacted by the presence or absence of the polymorphisms [Fig. S1(D)].

**FIG. 2.**
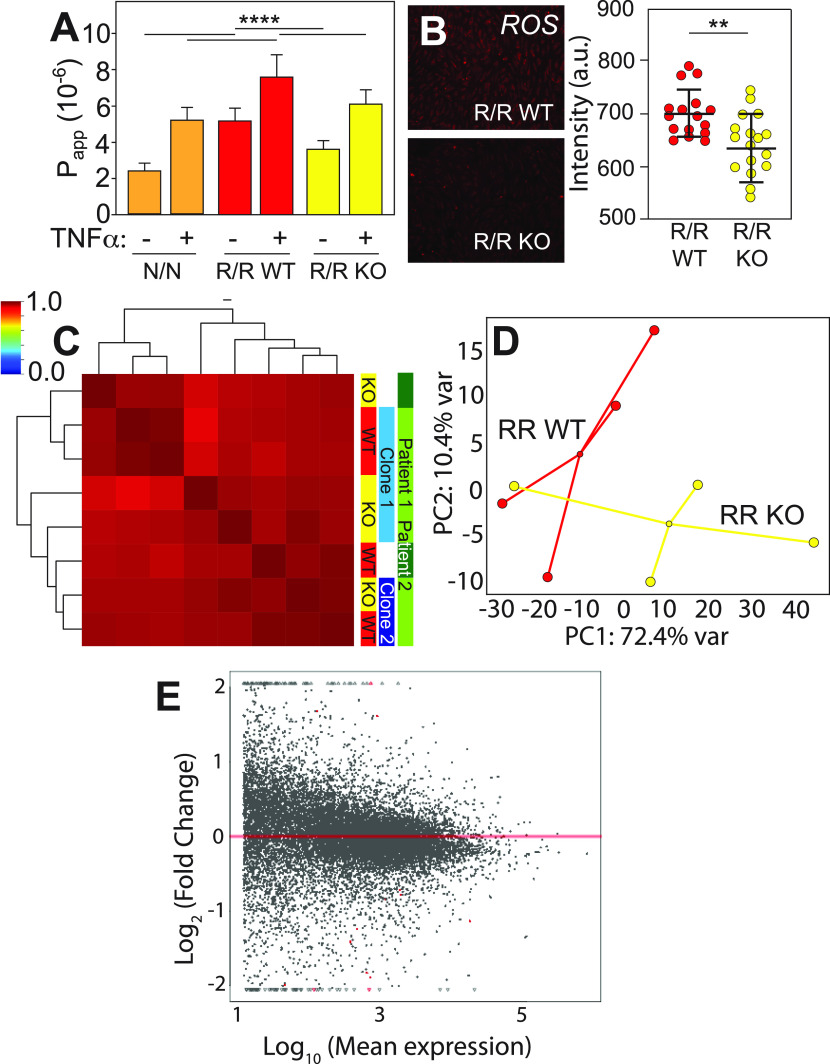
Inflammatory cytokine impact on the EC barrier function depends on risk haplotype. (a) Apparent permeability is plotted as a function of risk haplotype and the presence or absence of TNFα for 12 h at 1 ng/ml concentration. ****p < 0.0001 for indicated comparisons via one way ANOVA with multiple comparisons Tukey test. n = 18–24. (b) Representative immunofluorescence images stained for ROS for the indicated risk haplotypes. At right, average cell intensity is quantified for n = 16–17 cell fields of view over triplicate experiments for R/R WT and KO, respectively. **p < 0.01 for indicated comparisons via the unpaired t-test. (c) Pearson's correlation plot is shown based on the whole transcriptome sequenced from patient iPSC-derived ECs treated with TNF and of the haplotypes indicated at right. Dendrogram indicates sample clustering; color map is plotted between 0 and 1 as consistent with other figures. (d) A 2D PCA plot is shown for transcriptomes from R/R WT (red) and R/R KO (yellow) ECs; patient origin is noted by an outline around each data point. The contribution of each PCA axis is noted alongside the axis itself. **(E)** Mean transcript expression is plotted as a function of the log ratio of change between WT and KO for all transcripts. Data in red represent genes with statistically significant (p < 0.1) differential expression based on DESeq2.

Morphological and functional assessments suggest that maturity is haplotype independent, but to ensure that this extends to the transcriptional level, we performed RNA-sequencing on iPSC-ECs, specifically of R/R WT and KO lines from two patients (C512 and C021), i.e., two clones from one patient (R/R WT clones 1–5 and 2–3 and isogenic R/R KO clones 1–9 and WB46) and one from the second patient (R/R WT clone ED2–70 and R/R KO clone ED2–65), and two differentiation batches of one isogenic clone pair (e.g., 1–5 and 1–9). A Pearson correlation plot of the entire transcriptome showed greatest clustering by patient and then by clone with consistent clustering of haplotype pairs with multiple differentiations under static conditions [Fig. 1(b)]; indeed, 3D principal component analysis (PCA) of the transcriptome groups by haplotype revealed little overall sorting [Fig. 1(c)]. Closer inspection of transcriptional differences indicated only 13 genes were differentially expressed genes (DEGs) for WT vs KO, i.e., those with an adjusted p-value threshold of 0.1 and an expression difference of >2-fold. However, when including genes with statistically different expression regardless of magnitude (86 total) with the same p-value threshold, we observed small fold-changes under static conditions [Figs. 1(d) and 1(e); supplementary Table 1]. Consistent with these observations, the most significant 20 gene ontology (GO) terms via Fisher's exact test included broad cardiovascular response categories [Fig. 1(f), red; supplementary Table 2]; those terms related to blood flow and forces (open arrowheads) and to inflammation (closed arrowheads) are highlighted and suggest that they are possible signaling mechanisms.

### Inflammatory signaling impairs iPSC-EC function but not transcription based on haplotype

The presence of inflammation GO terms and prior implication of inflammation in 9p21 pathogenesis^5^ led us to first assess the role that inflammatory cytokines, e.g., TNFα, could have on iPSC-EC haplotype-dependent function. iPSC-ECs cultured in 2D on transwell inserts were challenged after monolayer formation by exposure to TNFα, and apparent permeability was assessed. We found that risk cells were the most permeable with highest transmission of a 70 kD fluorescent dextran between chambers; removal of the locus partially restored barrier function [Fig. 2(a), red vs yellow]. Treatment with TNFα for 12 h increased permeability independent of haplotype, but cells containing the risk haplotype remained most permeable with removal of the locus partially restoring function relative to non-risk cells [Fig. 2(a), +TNFα]. GO terms for untreated cells were suggestive of a mechanism involving reactive oxygen species (ROS) secretion, and immunofluorescent assessment indicated reduced signaling when the locus was deleted [Fig. 2(b)]. These data suggested that the haplotype could be mediating transcriptional regulation induced by stress, e.g., inflammation, as we previously observed in other cell types with stress.^11,12^

To further understand the extent that TNFα caused haplotype-dependent transcriptional changes, we performed RNAseq on TNFα-treated iPSC-ECs. While the transcriptomes of unstimulated cells were sorted based on patient, clone, and then haplotype, a Pearson correlation plot of all transcripts from TNFα-treated cells indicated only subtle variation [Fig. 2(c)], consistent with functional observations, and not by patient or clone as with untreated cells in Fig. 1. While 2D PCA does indicate modest haplotype clustering [Fig. 2(d)], TNFα treatment resulted in fewer DEGs when comparing WT to KO cells [Fig. 2(e), red] to the same comparison with untreated cells; there were no statistically significant GO terms with this dataset. The lack of transcriptional changes with TNFα treatment while simultaneously having observed permeability changes suggested that regulation by the haplotype might not be transcriptional, unlike what we previously observed in other cell types when stressed.^11,12^ Since prior studies examined mechanical stresses, we next sought to understand how and to what extent iPSC-ECs respond in a more physiologically relevant niche to fluidic stress.

### Acute shear stress impairs iPSC-EC function via haplotype-based transcriptome regulation

**FIG. 3.**
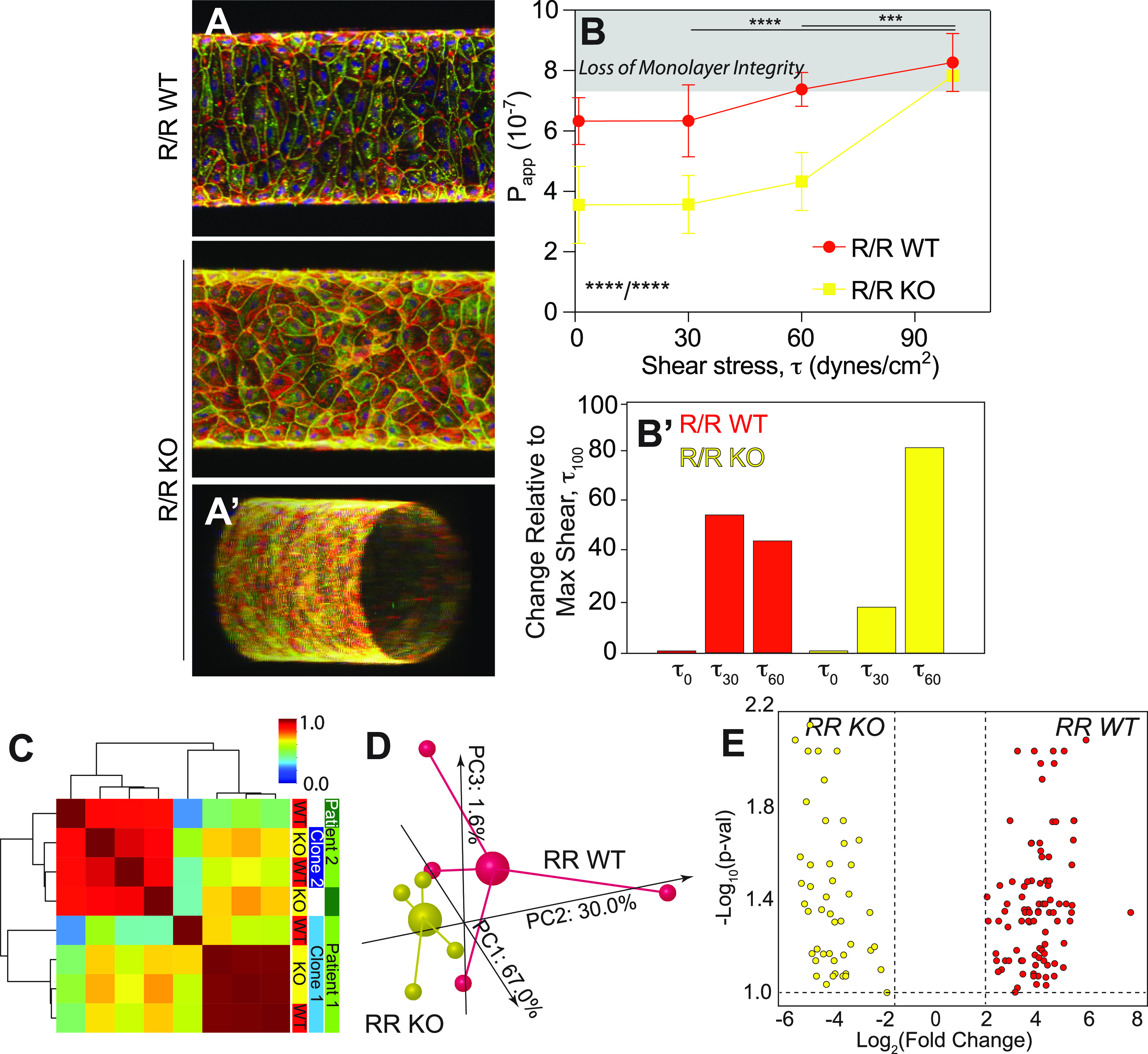
Exposure to acute shear reduces the EC barrier function for the Risk Haplotype. (a) Representative images of iPSC-ECs cultured in cylindrical vessels for 7 days and stained for ZO1 (green), F-actin (red), VE-cadherin (yellow), and nuclei (blue). R/R WT (top) and R/R KO (bottom) are shown with the latter including an oblique view (A′) to illustrate patency. (b) Apparent permeability, measured by dye exclusion assay, for R/R WT (red) and R/R KO (yellow) increases with acute exposure to shear. ****p < 0.0001 for shear and haplotype based on one way ANOVA with multiple comparisons Tukey test; ***p < 0.001 and ****p < 0.0001 for shear and haplotype comparison based on two-way ANOVA with multiple comparisons Tukey test. Note that in panel B′, the percent change in slope relative to maximum acute shear is plotted (i.e., the percent difference in the average values), highlighting the early onset of monolayer integrity loss as noted in Fig. S3F. n = 3–6 per haplotype and shear stress. (c) Pearson's correlation plot is shown for patient-derived iPSC-ECs based on the whole transcriptome sequenced of the haplotypes indicated at right. Dendrogram indicates sample clustering; color map is plotted between 0 and 1 as consistent with other figures. (d) Independent of patient and clonality, a 3D PCA plot is shown for all transcriptomes from R/R WT (red) and R/R KO (yellow) ECs cultured in the vessel geometry for 6 days followed by acute shear stress for 24 h. Individual samples are shown as smaller spheres, whereas sample averages are indicated by a larger sphere noting its linkage via identically colored lines. The contribution of each PCA axis is noted alongside the axis itself. (e) Volcano plot of 135 DEGs for transcriptomic comparisons of R/R WT (red) and R/R KO (yellow) ECs identified by DeSeq2 with an adjusted p-value threshold of 0.1. Colors indicate in which haplotype were the genes upregulated.

Endothelial cells are constantly exposed to shear stress *in vivo*. Under these conditions, the increased susceptibility to CAD due to 9p21 risk SNPs *in vivo*^22–24^ and subsequent mechanisms may be more pronounced in iPSC-ECs when exposed to shear stress. Thus, we constructed a microfluidic device consisting of a single-channel 3D microvessel [Fig. S3(A)], which exposes cells to laminar shear stress based on Poiseuille flow.^25^ iPSC-ECs were seeded within the media-filled collagen scaffold and allowed to form a monolayer within the microvessel channel under <1 dynes/cm^2^ fluid flow [Figs. 3(a) and 3(a′); Figs. S3(B)–S3(D)]; this simple design enabled us to perfuse vessels with 70 kD fluorescent dextran and measure apparent permeability in 3D via radial diffusion through the microvessel monolayer outwards to the lumen [Fig. S3(E)]. For iPSC-ECs derived from R/R WT and R/R KO lines, we measured permeabilities when exposed to a range from 1, 30, 60, or 100 dynes/cm^2^ for 24 h. Overall, both risk haplotype and shear stress were statistically significant variables by two-way analysis of variance (ANOVA) (p < 0.0001). More specifically, at physiological shear (<30 dynes/cm^2^),^26–28^ apparent permeability was significantly increased for the risk haplotype compared to their KO isogenic counterpart microvessels. However, permeability was insensitive to changes in shear in this range [Fig. 3(b)]. On the other hand, above 30 dynes/cm^2^, apparent permeability increased more at intermediate shear for R/R WT than their isogenic counterpart R/R KO cells [Fig. 3(b′)]; in this pathological shear range for CAD,^28^ monolayer integrity was lost [Fig. S3(F)]. Much above this range at 100 dynes/cm^2^, apparent permeability differences were lost (p = 0.99) as was monolayer integrity for both haplotypes. These data suggest that the microvessels of risk haplotype are more susceptible to vessel dysfunction at pathological shear but that deletion of the haplotype rescues the phenotype.

To determine the transcriptional changes associated with the onset of risk haplotype dysfunction, we performed RNA-sequencing on iPSC-ECs exposed to 30 dynes/cm^2^, which is the point of pathological shear onset for CAD,^28^ where we observed significant differences in permeability despite having an intact monolayer (avoiding selection bias). A Pearson correlation plot of the entire transcriptome under acute shear showed stronger clustering by patient and clone than haplotype [Fig. 3(c)] vs static conditions. However, when clustering by haplotype alone in a 3D PCA plot and unlike with static conditions, we still observed substantial separation based on the entire transcriptome [Fig. 3(d)]. These data suggest that shear stress actives haplotype regulation on the transcriptome. What could result from such regulation is an increase in the number of DEGs between WT and KO cells; thus, we also examined DEGs under shear, defined here as an adjusted p-value of 0.1 and an expression difference of >2-fold. With these cutoffs, we detected 135 DEGs between WT and KO cells as a result of shear (supplementary Table 3). The number of DEGs when cultured with shear is nearly 10-fold higher [Fig. 3(e)] than without.

**FIG. 4.**
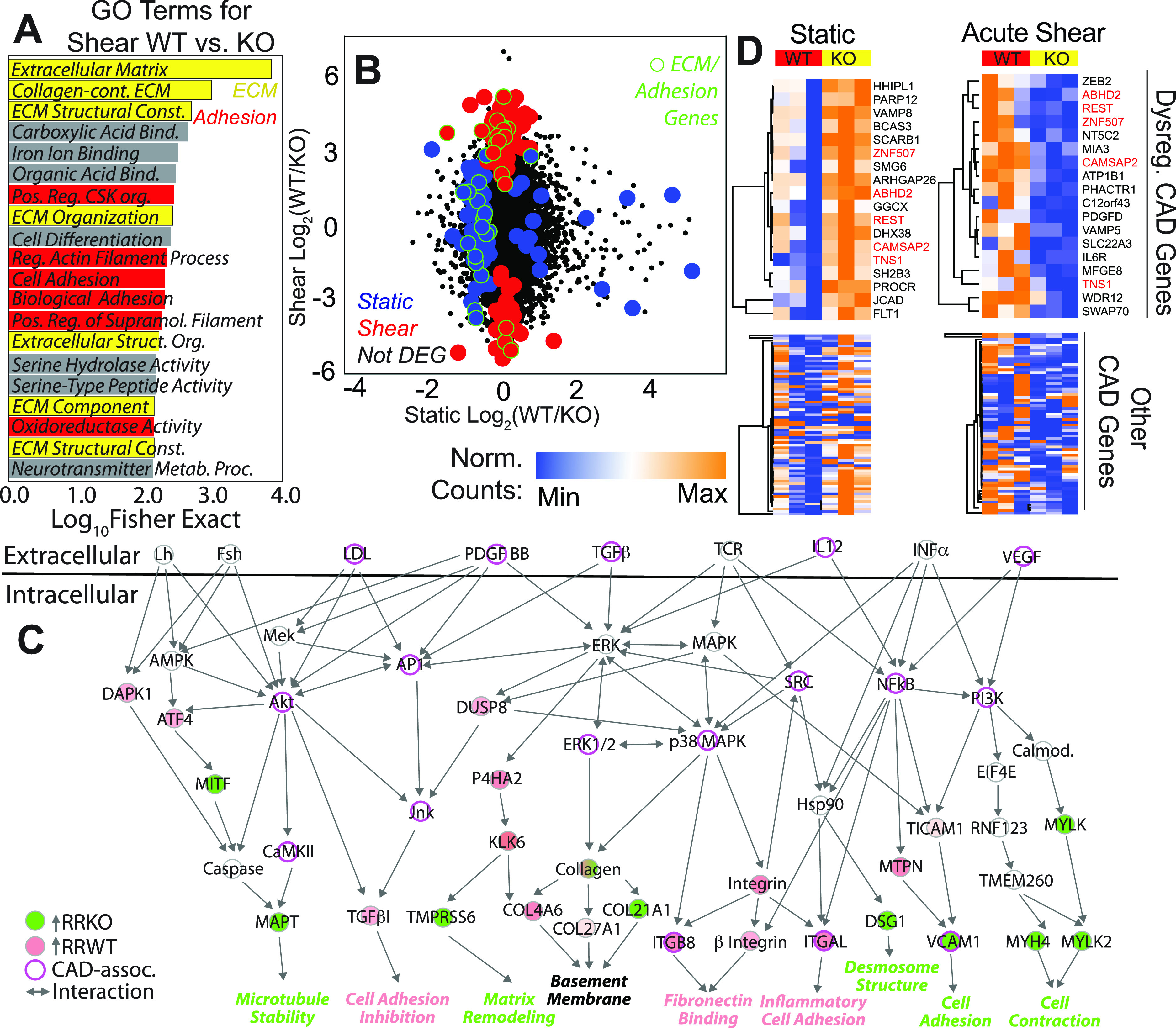
Shear-mediated haplotype regulation affects adhesion and CAD transcriptional signatures. (a) Top 20 statistically significant GO terms for the R/R WT vs KO comparison from the TopGO library. Terms associated with ECM (yellow) and adhesion (red) are indicated. (b) Scatter plot is shown for the log_2_ fold change of the R/R WT to KO ratio for each transcript of iPSC-ECs from sheared and static conditions. Black data points indicate non-DEGs, blue indicates DEGs for static only, and red indicates DEGs for shear only. Data points outlined in green represent those associated with ECM or adhesion GO terms. (c) Ingenuity analysis identified several differentially expressed genes in signaling pathways linked to CAD via the IPA database. Coloring of gene nodes indicates upregulated expression relative to R/R WT (red) or R/R KO (green). CAD associated genes via IPA database and previous literature are shown with purple highlighted outline. (d) Heatmaps showing gene expression for non-zero genes whose loci have been linked to CAD via the CARDIoGRAMplusC4D study. Values shown were computed by DESeq2's median of ratios. Top heatmaps show statistically significant CAD associated genes between R/R WT (red) and KO haplotypes (yellow). Bottom heatmaps show expression of the other genes identified from the CARDIoGRAMplusC4D study (bottom). Genes sorted into the top heatmaps had p < 0.1 for unpaired t-tests. Left and right heatmap columns are for iPSC-ECs under static or acutely sheared conditions, respectively. DEG names are shown to the right of top heatmaps with red highlighting, indicating common genes between conditions.

**FIG. 5.**
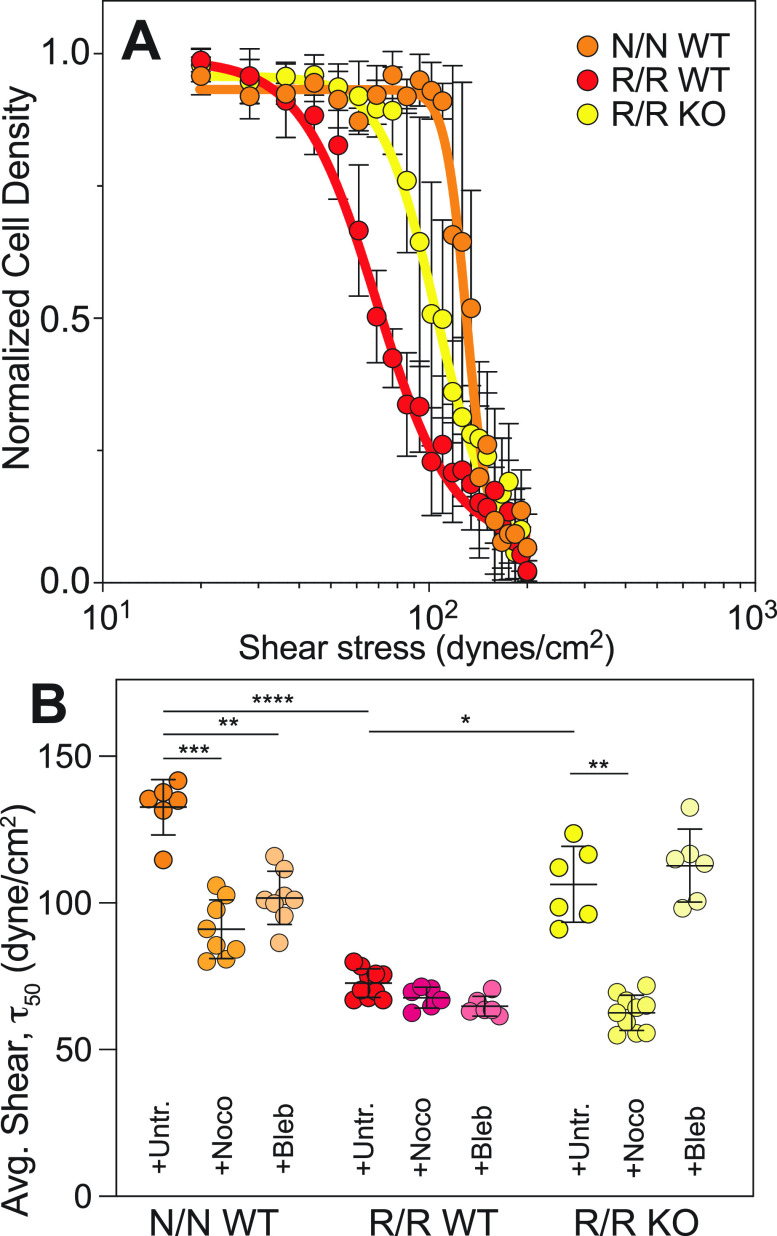
Haplotype regulates adhesion via microtubule cytoskeleton. (a) Plot of cell density vs fluid shear stress for each iPSC-EC haplotype measured in the converging parallel plate flow chamber. Data are shown from individual chambers and multiple technical replicates of the same cell line. Lines in the plot indicate sigmoidal fits of the data to determine τ_50_, the shear stress where 50% of the cells detach. (b) The effect of microtubule depolymerizing drug nocodazole (+Noco) and myosin inhibitor blebbistatin (+Bleb) on τ_50_ is plotted for the indicated haplotypes. *p < 0.05, **p < 0.01, ***p < 0.001, and ****p < 0.0001 for drug comparisons within each haplotype based on one way ANOVA with multiple comparisons Tukey test.

To investigate potential shear-mediated haplotype-specific mechanism, we next plotted the most significant gene ontological terms by Fisher's exact test, finding that of the top terms, those associated with extracellular matrix (ECM) and cell adhesion were most abundant [Fig. 4(a); supplementary Table 4], suggesting that loss of monolayer integrity at even higher shear could be a result of haplotype-specific transcriptional silencing of cell adhesion receptors. To further confirm the effects of shear on adhesion and ECM and eliminate underlying variance from the haplotype alone, we compared transcriptome differences between isogenic WT and KO pairs in shear and static conditions. As shown in Fig. 4(b), overall variance was greater with shear, hence the elongated distribution. Genes differentially expressed in the sheared WT/KO ratio (red) tended to show larger and more numerous effects than in static conditions (blue). This was especially pronounced for transcripts associated with adhesion and ECM (green outlined data); genes within these ontologies largely changed from down- to up-regulated with shear, perhaps to counteract haplotype effects resulting in monolayer loss. However, to interpret these data in a network context, we next mapped isogenic WT and KO pairs in shear vs static conditions onto common CAD pathways present in ingenuity pathway analysis (IPA). A diagram of signaling connections [Fig. 4(c)] illustrates potential haplotype signals; pathways that positively regulate cell adhesion appear to increase with shear in KO cells (green), whereas those that negatively regulate cell adhesion appear increased in WT cells (pink). Therefore, we performed a comparison of these data to known CAD genes identified from meta-analyses, e.g., the CARDIoGRAMplusC4D consortium^29^ as well as others.^30,31^ From these studies, 91 genes associated with 88 loci are suggested to be dysregulated in CAD. Genes differentially expressed in the presence of the risk haplotype, that appeared to be shear-sensitive, and that overlap with CAD-associated genes were found on multiple chromosomes indicating wide haplotype regulation; in general, weakly upregulated genes in static conditions for R/R KO reversed and became more strongly upregulated in R/R WT under shear [Fig. 4(d); supplementary Table 5]. In addition to global CAD risk activation upon exposure to pathologically high shear, several CAD-associated genes were found in both static and shear conditions with opposing expression. TNS1 and CAMSAP2 [Fig. 4(d), red], for example, relate to cellular mechanical functions such as cytoskeletal stability, which aligns with previous ontologies and IPA. With several transcriptional indicators of cytoskeletal response to shear stress, we sought to confirm their influence on function via shear assays in a converging parallel plate flow chamber. This assay applies a family of shear stress (Fig. S4) to cells to determine the shear stress at which 50% of population detaches from the coverslip, i.e., τ_50_. Consistent with transwell assays, N/N WT and R/R KO were more adherent than their R/R WT counterpart that contains the risk locus [Fig. 5(a)]. When treated with nocodazole to perturb microtubule assembly, N/N WT and R/R KO cells detached from the substrate at similar levels to R/R WT cells. Addition of blebbistatin treatment to inhibit myosin contraction showed no significant changes in τ_50_ for the isogenic comparison of R/R WT and R/R KO cells, though N/N WT cells had decreased adhesion [Fig. 5(b)]. These data validate microtubule transcriptional changes and suggest that their influence on downstream networks may be the likely drivers of haplotype-specific functional differences. Overall, these data suggest for the first time that mechanical regulation of endothelial cell function could be haplotype-specific and modulated by high shear stress.

## DISCUSSION

While correlations found with genome wide-association studies have identified many SNPs related to disease,^3,4^ mechanisms for those in non-coding loci are not well understood, e.g., the non-coding 9p21 locus and risk of endothelial permeability and disease. Difficulties could result from the linkage disequilibrium of the locus' polymorphisms,^5^ multiple, overlapping disease risk factors,^32^ contradictory behavior of animal models,^9,33^ or the requirement of a covariant stressor.^11,12^ Here, we found that iPSC-derived endothelial monolayers had increased apparent permeability under static conditions with the risk haplotype and when stressed with an inflammatory cytokine. More strikingly, when placed in a microvessel under high, acute shear stress consistent with CAD,^26–28^ endothelial cells with the risk haplotype exhibited increased apparent permeability compared to KO cell types in part because of the loss of monolayer integrity, similar to the leaky vasculature observed clinically with CAD.^34^ Only at supra-pathological shear stress,^28^ e.g., 100 dynes/cm^2^, did KO cell monolayer lose integrity and approach the same permeability as their risk WT counterparts. Detailed analyses of known CAD genes^29–31^ and pathways further implicate adhesion changes as a potential mechanism for haplotype-mediated changes. These results suggest that mechanical stress pathway(s) can induce EC dysfunction more than inflammatory cytokine TNFα and that shear stress causes dysfunction in a haplotype-dependent manner via modulating cell-cell and cell-ECM adhesion gene expression. Alongside our prior work,^12^ these data are generally supportive of the framework where environmental interactions act in concert with genetic variants to induce iPSC-derived progeny responses similar to *in vivo* assays, which may be impractical or impossible in common laboratory animals.^7–9^

The effects from both excessive wall shear stress and inflammation are well known in CAD, e.g., EC barrier function is highly susceptible to inflammatory cues.^17^ For the 9p21 locus, cytokines such as TNFα and IL-6 are higher in patients with the risk haplotype.^35^ Interferon signaling is also a known regulator of this locus' activity,^5^ which implies that in the presence of the risk haplotype, CAD progression might also occur indirectly via transcriptional regulation. Conversely, CAD risk factors also include the presence of turbulent flow or high magnitude oscillatory flow,^20^ both of which can damage the endothelium.^19^ Until this study, however, it was unclear if high wall shear stress could regulate the activity of a non-coding locus in and of itself and similarly influence cell function. Here, we found that not only was the TNFα effect marginal on iPSC-derived ECs, it was insensitive to the risk haplotype; but also minor differential transcriptional regulation was detected as a function of haplotype. However, significant functional and transcriptional changes occurred when cells were perfused slowly in a microvessel model for an extended period and then exposed to acute, high wall shear stress. The implication from these data is that transcription changes with shear mirror some gene activation patterns found in the meta-analyses of CAD transcriptional differences.^29–31^

High shear flow resulted in increased EC permeability and diminished monolayer integrity, which are clinical hallmarks of disease.^28,36^ At shear stresses just prior to EC detachment, we found a switch from down- to up-regulation of a subset of cell adhesion genes, perhaps to counteract haplotype-specific loss of cell-cell regulators, e.g., risk WT cells exhibited a 25-fold reduction in vascular cell adhesion molecule (VCAM). Indeed, both IPA and GO analyses showed knockout cells expressing pathways related to contractility, cell-cell adhesion, and ECM; conversely, the risk haplotype expressed inhibitory pathways. Although a 9p21-specific relationship with shear and adhesion has not been previously documented, stress-mediated monolayer disruption in ECs has been noted both *in vitro* and *in vivo*^11,16,19^ and suggests some fidelity between model observations and clinical presentation. We also found that modulation of microtubule assembly caused haplotype-specific loss of adhesion and monolayer disruption under shear stress, e.g., R/R WT, and literature suggests that this could hinder flow-induced dilation of artery vessel.^37,38^ In parallel, cytokines are classically known to activate endothelial cell expression of leukocytes adhesion receptors,^39^ which facilitates their migration into subintimal spaces. We observed similar changes in risk WT ECs, albeit when exposed to acute shear stress. Leukocyte infiltration can be extremely detrimental to established disease,^36,39^ hence concern from a 13-fold upregulation of integrin α_L_ with risk haplotype. When present, leukocyte signals^40^ have even mirrored the cell erosion observed here at high shear stress. In addition, AP-1 or NF-kB binding mechanisms—pathways identified to be related to the significantly different biomechanical cell structures, notably here in microtubules and adhesion—have been found to be influenced by several other genetic variants.^41^ Differential endothelial enhancer activity in response to unidirectional shear has also been found to correlate with GWAS identified risk loci SNPs at 1p32.3, which mirror the endothelial mechanical deficits found in the R/R WT ECs.^42^ With this in mind, there is a high likely hood that the genotypic expression discrepancies in the R/R WT ECs caused by the risk haplotype presence are related to the mechanotransduction deficits of adhesion response to fluidic shear. Thus, adhesion modulation appears in multiple mechanisms of CAD, and 9p21 could play a part in that mechanism.

Finally, the combination of iPSC-derived ECs and a 3D microvessel model afforded us a unique opportunity to study the 9p21.3 haplotype in a more appropriate setting *in vitro*; as previously noted, particular loci are found only in evolutionary relatives of humans and not in common laboratory species,^7,8^ complicating disease models. Patient-derived iPSCs have been used as an alternative,^10^ but standard culture conditions may not induce the regulation one intends to study owing to a locus' variable penetrance or its indirect effects on disease.^43^ Conversely, significant effort over the past two decades has resulted in a wide variety of biomaterials and fabrication methods to create the appropriate context in which to test a hypothesis about genetic regulation of disease. Our own recent evidence suggests that microenvironmental changes are necessary, e.g., stiffening of the niche,^12^ to induce expression of lncRNA in the risk haplotype. Only at that point does their presence cause asynchronous contraction of iPSC-derived cardiomyocytes. Similar time-dependent stiffness changes can induce subtle but more physiologically appropriate initiation of epithelial-to-mesenchymal transition^44,45^ or loss of progenitor cell potency.^46^ The microfluidic vessel used in this study similarly induced haplotype regulation upon exposure to acute, high shear stress. However, CAD flow patterns are exceedingly complex^28,36^ and further refinement of our model, e.g., inclusion of varied flow patterns such as unstable flow, disturbed flow, or pulsatile flow, or variations in vessel geometry such as in flow separation or vessel bifurcations to emulate atherogenic factors could better refine our system further and perhaps uncover additional regulation of the niche on the risk haplotype.

## METHODS

### Endothelial cell (EC) differentiation

iPSCs were differentiated into ECs using an established protocol^21^ [Fig. S1(B)]. Briefly, iPSCs were plated at densities ranging from 32 000 to 52 000 cells/cm^2^ on day 0. Cell media was then changed to N2B27 + CHIR media on day 1. Cell media was changed to Stempro + Forskolin + VEGF on day 4. Cell media was again changed on day 5. Cells were sorted on day 6 using flow assisted cell sorting (FACS) for VE-Cadherin immunostaining and plated for 7 days for endothelial maturity.

### Immunofluorescence imaging

Cells were immersed in an ionic solution of 1 mM MgCl2 and 0.1% (w/v) Saponin for the duration of the immunostaining. Between each step, the sample was washed three times with 1 mM MgCl2 solution. Cells were introduced to only 0.1% (w/v) Saponin for 15 min before blocking with 2% goat serum solution for 30 min. Primary antibody solution was added into saponin and goat serum solution as noted before added to the sample for 2 h. These antibody solutions include VE-Cadherin 1:100 concentration (cell signaling D8752) and ZO1 1:200 (Abcam 221546). Secondary fluorescent antibodies were added 1:1000 to the samples in the goat serum + saponin solution for 45 min at room temperature. Additionally, rhodamine phalloidin may be added for actin visualization. DAPI dye was then added to the sample for 3 min at a 1:400 dilution. The sample was then prepared using fluoromount on a microscope slide for imaging [Fig. 1(a)]. Cells were imaged using Zeiss 780 confocal microscope.

### Circularity analysis

Endothelial cells were stained using immunofluorescent staining techniques described earlier. Samples were imaged using Zeiss 780 confocal microscope. Cell area and perimeter were measured using ImageJ software. Circularity was calculated using the cell area and perimeter measured [Fig. 1(b)].

### ROS detection assay

Mature and confluent endothelial monolayers were washed twice with PBS before being immersed in a 5 *μ*M dihydroethidium solution at 37 °C for 20 min. Samples were then washed twice with 1 mM MgCl_2_ solution before being fixed using 10% formaldehyde solution. Samples were then mounted and imaged as described above [Fig. 2(c)]. Cell nuclei intensity was then measured using ImageJ software.

### Dye exclusion assay

iPSC-derived endothelial cells were cultured on a transwell permeable support for 6 days. On the sixth day, cells were serum starved for 24 h before the start of the assay. In some sample groups, TNFα was added to the solution at 1 ng/ml in serum starve conditions 12 h before assaying. A 24-well plate fitting the corresponding permeable transport was filled with 600 *μ*l media solution for each well for each time point and permeable support. The top compartments of each permeable support were replaced with new media at the start of the assay also containing 0.2 mg/ml 70 kDa FITC-Dextran at the start of the assay. Over a period of one hour at 15-min intervals, the permeable support containing confluent endothelial cells was transferred from one well to a new well. Samples of each time point and each permeable support were then transferred to a 96 well plate for imaging in a Syngery 4 multi-mode microplate reader [Fig. 2(a)]. Using a FITC-Dextran standard, mass diffused over time and apparent permeability were calculated.

### RNA isolation

Cells were lysed with Trizol. Chloroform was added 1:5 to trizol and moved to Eppendorf microcentrifuge tubes. Samples were then spun down at 14 000 rpm for 15 min using an Eppendorf centrifuge 5424 R. Aqueous solution separated during centrifugation was removed from the top of samples and mixed 1:1 with isopropanol and made to sit for 10 min at 4 °C before centrifugation at 14 000 rpm for 10 min. The sample supernatant was then removed and replaced with 75% ethanol with diethylpyrocarbonate (DEPC) water before centrifugation at 11 000 rpm for 5 min. The supernatant was once again removed and the sample-containing Eppendorf tubes allowed to dry for 10 min. Sample pellets were then resuspended in DEPC water, and the RNA concentration was calculated using a Thermofisher Nanodrop 2000C. In the event of having small cell quantities to work with, a Qiagen miRNeasy kit was used for RNA extraction. The kit protocol was followed as instructed.

### Quantitative Polymerase Chain Reaction (PCR)

Cell-isolated RNA was made into cDNA for quantitative PCR analysis using the invitrogen superscript III reverse transcriptase kit. cDNA was aliquoted into a 384-well plate according to the number of primers being used and a mixture of primers, DEPC water, and power SYBR green PCR master mix was added to each sample aliquot. This 384-well plate was then read in the BioRad CFX 384 touch real-time PCR detection system.

### Creation of microvessel model

Glass-bottom petri dishes were used as the frame of the devices in a process outlined in Fig. S3. Holes of 0.64 and 1.27 mm were drilled into opposite sides of the petri dishes, directly above the bottom of the petri dish and perpendicular to the petri-dish wall. Blunt-end pins of 23 G and 18 G (0.25 in.′) were fit snugly into the drilled holes, making sure to not allow for unusual cracks or holes. A steel cylinder was then fit from one end of the petri dish to the other through the blunt end pins. PDMS with ratio of crosslinker 1:10 elastomer was then poured to fill the modified dish and allowed to solidify. PDMS was degassed prior to addition to the mold to ensure smooth interface. After the PDMS had solidified, PDMS was removed from the center of the modified dish using a scalpel according to the desired chamber dimensions. The blunt-end pins were allowed to extend beyond the wall of the chambers to disperse pressure away from the collagen-PDMS interface and instead further into the collagen gel and the lumen. The gel chamber is cleaned to remove any small PDMS residue. The seal of the bottom glass and the petri-dish were ensured intact and unbroken. Freshly made PDMS is then poured into a petri-dish lid according to the volume of the intended gel chamber, being sure to not create bubbles. The modified dish is then inverted and placed over the fresh PDMS in the lid to seal the gel chamber. The PDMS was not allowed to rise to the blunt-end pin openings of the gel chamber in order to allow for unobstructed inlet and outlets of the gel chamber. The modified dish is then allowed to rest for at least two days.

To create the gel scaffold within the microfluidic devices, all components, including the PDMS-dish case, the blunt-end pins, and any other framework supports, such as the microfluidic stand, were sterilized. The blunt-end pins were then fit into the appropriately sized holes in the PDMS-dish case. Any introduction of liquid to the device was then added via leur-lock syringe through the inlet only, which has a smaller diameter than the outlet, to minimize pressures within the gel chamber. The chamber is washed once with PBS and then coated with 0.1 mg/ml poly-D-lysine for 5 min before being washed out with PBS. This is set to dry overnight.

Type 1 collagen gel was used for the gel scaffolding for all experimentations within the microfluidic device. For a 6 mg/ml concentration collagen gel, 1 M NaOH at 0.024 the volume of collagen and 10× PBS at 0.1 the total volume were mixed together in a small petri dish on ice. Type 1 collagen gel, chilled on ice, was mixed well into the NaOH and PBS mixture until uniformity, avoiding bubble formations. Deionized water was added and mixed to fill the remaining total volume. The finished type 1 collagen gel mixture was then syringed into the microfluidic device from the inlet slowly, allowing for minimal bubble formation but before warming of the gel occurs, until the entire gel chamber is filled and halfway into the outlet blunt-endpin. A thin steel cylinder of 340 *μ*m diameter was then inserted through the inlet, and the outlet was then gently sealed with a male leurlock plug. The device was then heated at 37 °C in an incubator with the outlet side up for 2 h for gel solidification. 2 h after solidifying, the microfluidic device was removed from the incubator and the thin steel cylinder was removed gently from the device. The channel was then inspected for any obstructions and flow tested from outlet to inlet. The collagen gel is then crosslinked with a 20 mM genipin solution for 2 h at 37 °C. After 2 h, the microfluidic device was washed with PBS at room temperature. The device was washed with PBS overnight at room temperature to remove any residual genipin solution.

### Preparation and use of microvessel model

After overnight PBS wash, the microfluidic devices were coated along the lumen of the collagen scaffold with 0.1 *μ*g/ml fibronectin for greater cell attachment during seeding [Fig. S3(C)]. Each side was coated for an hour with fibronectin solution for even distribution on all sides of the microfluidic channel. With coating complete, the microfluidic device was perfused overnight with EGM2 media at room temperature to allow for displacement of PBS-scaffold liquid for cell nourishing media.

To seed cells into the microfluidic device, a device was placed at 37 °C in an incubator 30 minutes before seeding to warm up the media and device. Endothelial cells were lifted and prepared in solution at 10 × 10^6^ cells/ml in EGM2. 8 *μ*l of cell solution was then pipetted into the device through the inlet, allowing for the cell solution to flow through and into the lumen of the device, and then the microfluidic device was placed in the incubator for 5 min. After incubation, the device was reseeded with another 8 *μ*l of cell solution again and placed on another side of the device for 5 min in the incubator, and continuing for the other side and top of the device. This allowed for even distribution of cells along all sides of the device. After seeding all four sides of the device (top, sides, bottom), the microfluidic device was placed in the 37 °C incubator to allow for cells to strengthen attachment. One hour later, fresh media was added to the microfluidic device. 10 hours later, the microfluidic devices were connected to a peristaltic pump for long term perfusion. Media reservoirs were changed with fresh media every 2 days.

### Microvessel permeability assay

On the sixth day of pump perfusion, the microfluidic shear is increased to the desired shear stress magnitude and held for 24 h. After perfusion, microfluidic devices were gently removed from the pump and attached to a microscope slide. The microfluidic device was then mounted on the microscope platform of a Zeiss 780 confocal microscope. The image area was centered on the channel to allow for observation of diffusion. The microfluidic device was then perfused at approximately 0.4 *μ*l per minute with 0.2 mg/ml 70 kDa FITC-Dextran dye in EGM2 media over 30 min, with fluorescent images taken every 2-min [Fig. S3(E)]. The fluorescent intensity of a consistently standardized area around the microfluidic channel is then measured for each image using ImageJ software. Change in fluorescence intensity is calculated over time, which is then used to calculate apparent permeability of the microfluidic device based on channel dimensions.

### Converging parallel plate flow chamber

Geometry of the flow chamber was cut in Teflon tape sheets using the Silhouette Cameo 4 desktop cutter. Teflon tape designed cuts were then attached to glass slides and sterilized for cell seeding. iPSC-derived endothelial cells were cultured on Teflon taped glass slides for 6 days. On the sixth day, cells were serum starved for 12 h before the start of the assay. In some sample groups, nocodazole or blebbistatin was added to the solution at 0.66 or 10 *μ*M, respectively, in serum starve conditions. The plates were inserted into the converging parallel plate by fitting the glass slide into a silicone gasket and sealing the flow chamber using screws. Before images of the glass slides were taken under phase contrast before attaching the sealed flow chamber to a peristaltic pump for shearing, glass slides were sheared for 5 min using a PBS 4.5 mg/ml dextrose solution at 37 °C at the designated shear values before taking after images of the glass slides. The cellular detachment of each glass slide can then be calculated by the area coverage lost from the before to after images using ImageJ software.

### RNA sequencing

Isolation of RNA was conducted as described above. Samples were submitted to UCSD Institute for Genomic Medicine Sequencing Core. Total RNA was assessed for quality using an Agilent Tapestation 4200, and samples with an RNA Integrity Number (RIN) greater than 8.0 were used to generate RNA sequencing libraries using the TruSeq stranded mRNA sample prep kit with TruSeq unique dual indexes (Illumina, San Diego, CA). Samples were processed following manufacturer's instructions, modifying RNA shear time to 5 min. Resulting libraries were multiplexed and sequenced with 100 basepair (bp) paired end reads (PE100) to a depth of approximately 25 × 10^6^ reads per sample on an Illumina NovaSeq 6000. Samples were demultiplexed using bcl2fastq v2.20 conversion software (Illumina, San Diego, CA). Analysis was conducted using STAR, R, DeSeq2, and python software.

### Statistics

All experiments were performed using cells from three distinct differentiations with the number of technical replicates, n, indicated where appropriate. Bar graphs and scatter plots with individual data are represented as mean ± standard deviation. Statistical analyses were performed using GraphPad Prism5, the threshold for significance level at p < 0.05, unless otherwise noted, and are detailed in the corresponding figure legends.

## SUPPLEMENTARY MATERIAL

See the supplementary material for four figures, which further detail haplotype differences between lines and outline the methods used for the microfluidic vessel-in-a-dish and the converging parallel plate flow chamber models. Five supplemental tables are provided to list the differentially expressed genes (DEGs) and gene ontological (GO) terms from bulk RNA-sequencing of WT vs KO haplotype comparisons of differentiated iPSC-derived ECs with and without shear stress. Tables also list CAD-associated genes not dysregulated by the risk haplotype.

## Data Availability

The accession number for the RNA-seq data reported in this paper is GEO: 21738935. The data that support the findings of this study are available within the article and its supplementary material. Source data for the figures are available from the corresponding author upon reasonable request.
